# Assessment of the efficacy and prognostic value of 18F-FDG PET-CT using deauville 5-point scale and ΔSUVmax methods in diffuse large B-cell lymphoma patients

**DOI:** 10.1097/MD.0000000000044067

**Published:** 2025-09-05

**Authors:** Jian-Ping Li, Pei Wang, Fu-Fu Liu, Min Wu, Kan Deng, Xin-Kai Wang, Yong Wu

**Affiliations:** a Department of PET-CT Imaging Diagnosis, Shanghai Tianyou Hospital, Shanghai, China; b PLA Navy Specialty Medical Center, Shanghai, China; c Department of Nuclear Medicine, Xi’an Gaoshang Medical Imaging Diagnosis Center, Xi’an, Shaanxi, China.

**Keywords:** 18F, cell lymphoma, CT, Deauville 5, diffuse large B, FDG PET, point scale, prognostic value, ΔSUVmax method

## Abstract

Diffuse large B-cell lymphoma (DLBCL) requires accurate therapeutic response assessment. This study evaluates the efficacy and prognostic value of [18F] fluorodeoxyglucose positron emission tomography-computed tomography (18F-FDG PET-CT) using the Deauville 5-point scale and maximum standardized uptake value (ΔSUVmax) methods in DLBCL patients. A retrospective study was conducted from January 2021 to December 2022, including 60 DLBCL patients. Patients underwent baseline and interim PET/CT scans during chemotherapy, treated with the R-CHOP regimen. The Deauville 5-point scale visually assessed PET/CT results, while the ΔSUVmax method calculated the percentage change in SUVmax. Consistency with the Lugano criteria was evaluated using the Kappa statistic. Statistical analyses were performed with SPSS software (version 27.0, Chicago). Following 3 to 4 cycles of R-CHOP, 70.0% of patients achieved complete response, decreasing to 58.3% at 12 months. The Deauville 5-point scale classified 78.3% of patients as negative and 21.7% as positive, with moderate agreement with Lugano criteria (Kappa = 0.568, *P* < .01). The ΔSUVmax method identified 68.3% of patients as negative and 31.7% as positive, showing substantial agreement with Lugano criteria (Kappa = 0.728, *P* < .001). The ΔSUVmax method demonstrated higher sensitivity (81.33%), specificity (92.33%), negative predictive value (92.33%), positive predictive value (81.33%), and accuracy (90.68%). Both the Deauville 5-point scale and ΔSUVmax method are effective for evaluating interim therapeutic response and predicting 12-month outcomes in DLBCL patients. The ΔSUVmax method showed higher accuracy and predictive value. Integrating these methods into clinical practice can enhance patient prognosis and optimize treatment strategies.

## 1. Introduction

Diffuse large B-cell lymphoma (DLBCL) is the most prevalent subtype of non-Hodgkin lymphoma, accounting for approximately 30% to 40% of cases globally. Characterized by aggressive behavior and heterogeneous clinical manifestations, DLBCL presents significant challenges in diagnosis, treatment, and prognostication.^[1–3]^ The development of advanced imaging technologies has markedly improved our ability to assess disease status and therapeutic response, both of which are crucial for optimizing patient management and outcomes. [18F] fluorodeoxyglucose positron emission tomography-computed tomography (18F-FDG PET-CT) has become an essential tool in the staging and evaluation of DLBCL, capitalizing on the elevated glucose metabolism of malignant cells to precisely localize and quantify tumor activity.^[4,5]^ The role of 18F-FDG PET-CT in DLBCL has been well-established, demonstrating its utility in initial staging, treatment response evaluation, and relapse monitoring.

The Deauville 5-point scale and the percentage change in maximum standardized uptake value (ΔSUVmax) are 2 widely employed methods for interpreting 18F-FDG PET-CT results in DLBCL. The Deauville 5-point scale, a semiquantitative visual tool, categorizes ^18F-FDG uptake into 5 levels, providing a standardized framework for evaluating metabolic response. In contrast, the ΔSUVmax method quantifies the change in SUVmax between baseline and interim or end-of-treatment scans, offering a more objective assessment of metabolic response. Numerous studies have examined the prognostic value of 18F-FDG PET-CT in DLBCL, underscoring its role in predicting treatment outcomes and guiding therapeutic decisions. For example, early metabolic response, as assessed by interim 18F-FDG PET-CT, has been shown to correlate with progression-free survival and overall survival (OS) in DLBCL patients. Moreover, the combined use of the Deauville 5-point scale and ΔSUVmax methods may provide complementary insights, enhancing the prognostic accuracy of 18F-FDG PET-CT.

The primary objective of this study is to evaluate the efficacy and prognostic value of 18F-FDG PET-CT in DLBCL patients, utilizing the Deauville 5-point scale and ΔSUVmax methods. The integration of advanced imaging techniques with traditional clinical and molecular parameters holds promise for improving the precision and effectiveness of DLBCL management.

## 2. Methods

### 2.1. Study design

An exhaustive retrospective evaluation was conducted at our hospital to assess the efficacy and prognostic value of 18F-FDG PET-CT using the Deauville 5-point scale and ΔSUVmax methods in patients with DLBCL. The study period spanned from January 2021 to December 2022. A total of 60 patients, confirmed to have DLBCL through histopathological examination, were included in the study. The research methodology, intent, and protocols were meticulously designed following the Strengthening the Reporting of Observational Studies in Epidemiology (STROBE) guidelines and received approval from the Ethics Committee of our hospital.^[6]^

### 2.2. Inclusion and exclusion criteria

Patients included in this study were those diagnosed with DLBCL confirmed by histopathological examination. All patients underwent a baseline PET/CT scan before chemotherapy and an interim PET/CT scan during chemotherapy, specifically after the 3rd to 4th cycle of treatment. They were treated with the standard R-CHOP regimen or equivalent protocols and had not received any prior treatment for lymphoma before the baseline PET/CT scan. Additionally, all participants provided informed consent for the use of their medical records for research purposes. Patients were excluded if they had a history of other malignancies, had undergone surgical removal of lymphoma lesions before the baseline PET/CT scan, had a negative baseline PET/CT scan, or had uncontrolled or suspected infections, including chronic infections that were not manageable. Furthermore, patients with incomplete clinical, imaging, or follow-up data were also excluded from the study.

### 2.3. PET/CT scanning protocol

All patients underwent baseline and interim PET/CT scans using the same imaging equipment to ensure consistency. Among the patients, 28 underwent 18F-FDG PET/CT scanning 3 weeks after the completion of the third chemotherapy cycle, while the remaining 32 patients were scanned 3 weeks after the 4th chemotherapy cycle. Prior to the scans, patients were required to fast for at least 6 hours, ensuring optimal blood glucose levels below 11.1 mmol/L. They received an intravenous injection of 4.4 to 5.5 MBq/kg of 18F-FDG, with radiochemical purity exceeding 95%. The scans were performed 60 minutes postinjection, covering the area from the top of the skull to the upper third of the thighs. Low-dose CT was used for attenuation correction, and images were reconstructed using a 3-dimensional iterative algorithm before fusion with the CT images. Before study initiation, both nuclear medicine physicians underwent a calibration session in which they reviewed 10 representative PET-CT cases together to standardize application of the Deauville criteria. During the study, each physician assigned Deauville scores independently, blinded to the other reader’s results and to clinical outcomes. In cases of discordant scores (n = 6), a third senior nuclear medicine physician adjudicated, ensuring consistent final classification.

### 2.4. PET/CT image parameter measurement and analysis

Deauville 5-point scale: the Deauville 5-point scale was recommended for use at the inaugural International Lymphoma PET Symposium in 2009.^[7]^ This scale is employed to assess the therapeutic response at interim and end-of-treatment stages in lymphoma patients. The scoring is based on a visual comparison of the 18F-FDG uptake in the lesions to the 18F-FDG uptake in the liver and/or mediastinal blood pool. Scores range from 1 to 5, with scores of 1 to 3 considered negative and scores of 4 to 5 considered positive. The detailed scoring criteria are as follows:

- Score 1: no uptake above the background level.
- Score 2: uptake ≤ mediastinal blood pool.
- Score 3: uptake > mediastinal blood pool but ≤ liver uptake.
- Score 4: uptake moderately higher than the liver uptake.
- Score 5: uptake significantly higher than the liver uptake.
- Score X: new areas of uptake unlikely to be related to lymphoma.

ΔSUVmax method: The maximum standardized uptake value (SUVmax) represents the highest concentration of 18F-FDG uptake in DLBCL lesions. To calculate ΔSUVmax, the SUVmax values before and after treatment are compared. If residual lesions are present in the interim PET/CT scan, the SUVmax of the residual lesions is measured, even if the site differs from the baseline PET/CT scan. The highest uptake value at the residual site is recorded.^[8]^ If no lesions are detected in the interim PET/CT scan, the SUVmax is measured in the same region as in the baseline PET/CT scan. The reduction rate of SUVmax between the baseline and interim PET/CT scans is calculated as ΔSUVmax%. This percentage reduction is determined using the formula: ΔSUVmax% = (SUVmax_baseline - SUVmax_interim)/SUVmax_baseline × 100%.

### 2.5. Data collection and follow-up

Clinical and imaging data were collected from the baseline PET/CT scans of all patients. The data included age, gender, 2014 Lugano staging, Deauville 5-point scale scores, and SUVmax values from both the baseline and interim PET/CT scans. Follow-up information was gathered from inpatient and outpatient records, as well as telephone interviews. Disease control status was assessed over a 12-month period from the start of chemotherapy, based on the 18F-FDG PET/CT findings.

### 2.6. Evaluation of treatment response

Patients were categorized into 4 response groups – complete response (CR), partial response (PR), stable disease (SD), and progressive disease (PD) – using the 2014 Lugano criteria for lymphoma response assessment as the “gold standard.”^[9]^ For analysis purposes, PR, SD, and PD were grouped as non-CR (non-CR), facilitating a detailed evaluation of patient outcomes and treatment efficacy.

### 2.7. Statistical analysis

Statistical analyses were performed using SPSS software (Version 27.0, Chicago). Categorical variables were summarized as frequencies and percentages. The χ² test or Fisher exact test was used to compare categorical variables between groups, depending on the data distribution and sample size. Receiver operating characteristic (ROC) curves were generated to identify the optimal cutoff value for the ΔSUVmax% parameter, critical for distinguishing between different response categories. The area under the ROC curve (AUC) was calculated to assess the diagnostic performance of the ΔSUVmax% parameter. The agreement between the Deauville 5-point scale, ΔSUVmax method, and the Lugano evaluation criteria was evaluated using the Kappa statistic, which measures inter-rater agreement for categorical data, correcting for chance agreement. A Kappa value of 0 indicates no agreement, while a value of 1 indicates perfect agreement. A *P*-value of <.05 was considered statistically significant for all tests.

## 3. Results

### 3.1. Evaluation of patient responses using Lugano criteria

Following 3 to 4 cycles of R-CHOP chemotherapy, the Lugano evaluation criteria were applied to assess patient responses. Out of the 60 patients, 42 (70.0%) achieved a CR, indicating no detectable disease activity. Conversely, 18 patients (30.0%) did not achieve CR (non-CR), which included those with PR, SD, or PD. At the 12-month follow-up post-chemotherapy initiation, the response rates were reassessed. The number of patients achieving CR decreased slightly, with 35 patients (58.3%) maintaining CR status. In contrast, 25 patients (41.7%) were classified as non-CR.

### 3.2. Interim PET/ct parameters using Deauville 5-point scale for evaluating therapeutic response

The Deauville 5-point scale was used to assess interim PET/CT scan results. According to this scale, 47 patients (78.3%) were classified as negative, indicating no significant residual disease activity, while 13 patients (21.7%) were classified as positive. The consistency between the Deauville 5-point scale and the Lugano evaluation criteria was evaluated using the Kappa statistic, yielding a Kappa value of 0.568 (*P* < .01). This indicates a moderate agreement between the Deauville 5-point scale and the Lugano criteria, suggesting that the Deauville 5-point scale is a reliable method for interim assessment of DLBCL.

### 3.3. Interim PET/CT parameters using ΔSUVmax method for evaluating therapeutic response

**Figure 1.**
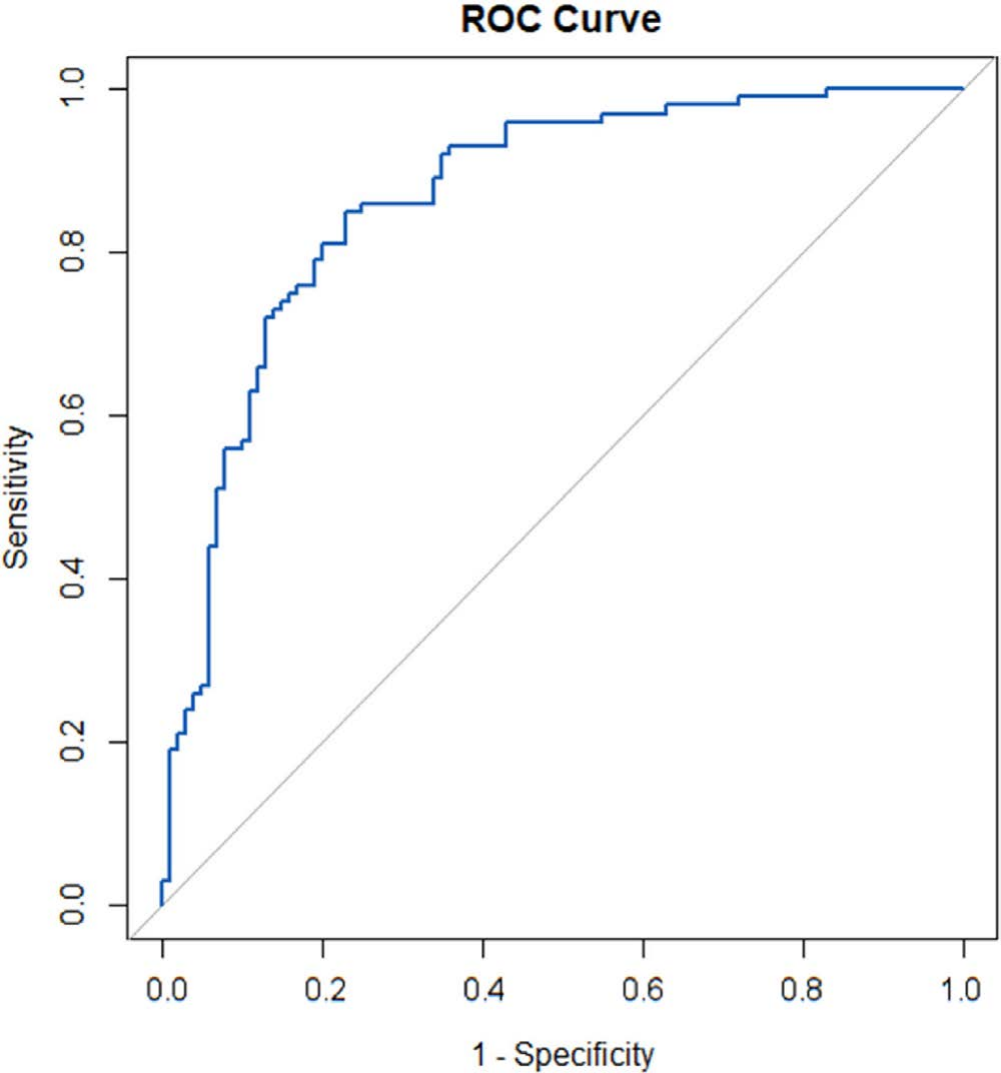
Receiver operating characteristic curve for evaluating therapeutic response using the ΔSUVmax method. ROC analysis of ΔSUVmax% in distinguishing Lugano-defined CR from non-CR after 3 to 4 cycles of R-CHOP. The AUC is 0.866 (*P* < .001). At the optimal ΔSUVmax% cutoff of 78.58%, sensitivity (true positive rate) is 81.33% (identifying CR patients correctly), and specificity (true negative rate) is 92.33% (identifying non-CR patients correctly). Each point on the curve represents a different ΔSUVmax% threshold, plotting sensitivity versus 1 – specificity. ΔSUVmax = maximum standardized uptake value, AUC = area under the curve, CR = complete responders, ROC = receiver operating characteristic.

The optimal cutoff value for ΔSUVmax% was determined using ROC curve analysis, with the AUC calculated at 0.866 (*P* < .001, Fig. 1). The optimal cutoff value for ΔSUVmax% was established at 78.58%. Patients with a ΔSUVmax% >78.58% were considered negative (41 patients, 68.3%), indicating a significant reduction in metabolic activity, while those with a ΔSUVmax% of 78.58% or less were considered positive (19 patients, 31.7%). The consistency between the ΔSUVmax method and the Lugano evaluation criteria was also assessed using the Kappa statistic, resulting in a Kappa value of 0.728 (*P* < .001). This indicates a substantial agreement between the ΔSUVmax method and the Lugano criteria, underscoring the effectiveness of ΔSUVmax% as a robust parameter for evaluating interim therapeutic response in DLBCL patients.

### 3.4. Comparison of diagnostic performance between Deauville 5-point scale and ΔSUVmax method

As detailed in Table 1, the ΔSUVmax method outperformed the Deauville 5-point scale on all metrics. Sensitivity was 81.33% versus 52.00%; specificity, 92.33% versus 80.35%; NPV, 92.33% versus 77.00%; PPV, 81.33% versus 80.35%; and overall accuracy, 90.68% versus 82.85%. These results indicate that ΔSUVmax provides a more reliable interim assessment of therapeutic response.

**Table 1:** Comparison of sensitivity, specificity, negative predictive value, positive predictive value, and accuracy between the deauville 5-point scale and ΔSUVmax method.

Method	Sensitivity (%)	Specificity (%)	NPV (%)	PPV (%)	Accuracy (%)
Deauville 5-point scale	52.00	80.35	77.00	80.35	82.85
ΔSUVmax method	81.33	92.33	92.33	81.33	90.68

ΔSUVmax = maximum standardized uptake value, CT = computed tomography, NPV = negative predictive value, PET = positron emission tomography, PPV = positive predictive value.

Deauville 5-point scale: a visual scoring system for PET/CT scan results in lymphoma patients.

ΔSUVmax: the percentage change in maximum standardized uptake value between baseline and interim PET/CT scans.

### 3.5. Predictive value of interim PET/CT parameters for 12-month disease remission

Deauville 5-point scale: according to the Deauville 5-point scale, patients classified as negative (47 patients) exhibited a significantly higher CR rate at 12 months compared to those classified as positive (13 patients). Specifically, the 12-month CR rate for negative patients was 65.96% (31 out of 47), whereas the CR rate for positive patients was only 30.77% (4 out of 13). This difference was statistically significant (*P* < .05), underscoring the utility of the Deauville 5-point scale in predicting long-term therapeutic outcomes.

ΔSUVmax Method: The ΔSUVmax method also demonstrated a significant predictive value for 12-month disease remission. Among patients classified as negative based on the ΔSUVmax method (41 patients), the 12-month CR rate was 78.04% (32 out of 41). In contrast, the 12-month CR rate for patients classified as positive (19 patients) was markedly lower at 15.79% (3 out of 19). This difference was statistically significant (*P* < .05), indicating that the ΔSUVmax method is a robust predictor of long-term remission in DLBCL patients.

## 4. Discussion

DLBCL is the most prevalent subtype of non-Hodgkin lymphoma, characterized by its aggressive nature and heterogeneous clinical presentation. Accurate assessment of therapeutic response is essential for optimizing treatment strategies and improving patient outcomes.^[9]^ 18F-FDG PET-CT has become an indispensable tool in this context, providing detailed insights into metabolic activity and disease burden. The Deauville 5-point scale and the ΔSUVmax method are 2 widely used techniques for interpreting PET-CT results. The Deauville 5-point scale, a visual assessment tool, categorizes ^18F-FDG uptake in lesions relative to the liver and mediastinal blood pool, offering a standardized approach for evaluating therapeutic response.^[10–12]^ However, despite its utility, the Deauville 5-point scale is inherently subjective and may lack sensitivity in detecting subtle metabolic changes. In contrast, the ΔSUVmax method quantitatively measures the percentage change in the maximum standardized uptake value (SUVmax) between baseline and interim scans, providing a more precise evaluation of metabolic response. This method is particularly effective in detecting minor changes in tumor activity, which could lead to more accurate prognostication.^[13,14]^

The ΔSUVmax method demonstrated superior sensitivity (81.33%) compared to the Deauville 5-point scale (52.00%), suggesting that it is more effective in accurately identifying patients with active disease during interim assessments. The enhanced sensitivity of the ΔSUVmax method can be attributed to its quantitative nature, enabling a more precise measurement of metabolic changes that may not be readily apparent through the visual assessment of the Deauville 5-point scale. Similarly, the ΔSUVmax method exhibited higher specificity (92.33%) than the Deauville 5-point scale (80.35%), indicating a better ability to correctly identify patients without disease. This increased specificity reduces the risk of false positives, thereby preventing unnecessary treatments and alleviating patient anxiety. Additionally, the ΔSUVmax method outperformed the Deauville 5-point scale in both negative predictive value (NPV) and positive predictive value (PPV), with NPVs of 92.33% and 77.00%, and PPVs of 81.33% and 80.35%, respectively. These findings highlight the ΔSUVmax method’s superior reliability in predicting both true negative and true positive cases. Overall, the accuracy of the ΔSUVmax method (90.68%) was higher than that of the Deauville 5-point scale (82.85%), reinforcing its superior diagnostic reliability.

When evaluating the predictive value of interim PET/CT parameters for 12-month disease remission, both the Deauville 5-point scale and the ΔSUVmax method exhibited significant predictive capabilities. However, the ΔSUVmax method demonstrated superior performance. Patients classified as negative by the Deauville 5-point scale had a 12-month CR rate of 65.96%, while positive patients had a CR rate of 30.77%. In contrast, for the ΔSUVmax method, the 12-month CR rate for negative patients was 78.04%, significantly higher than the 15.79% observed in positive patients. These differences were statistically significant (*P* < .05) for both methods. The higher predictive value of the ΔSUVmax method may be attributed to its ability to capture more subtle metabolic changes indicative of treatment response.^[15,16]^ By quantifying the percentage change in SUVmax, the ΔSUVmax method provides a more detailed assessment of tumor activity compared to the binary visual assessment of the Deauville 5-point scale. Several underlying mechanisms contribute to the superior performance of the ΔSUVmax method. First, its quantitative nature allows for a more accurate evaluation of changes in metabolic activity, which are crucial for monitoring treatment response.^[17,18]^ Tumors that respond well to chemotherapy typically exhibit a significant reduction in metabolic activity, reflected by lower SUVmax values. This reduction is more precisely captured by calculating the ΔSUVmax, which considers the relative change from baseline, offering a dynamic and individualized measure of treatment response.^[19,20]^

In contrast, the Deauville 5-point scale relies on visual assessment, which is susceptible to interobserver variability and may not detect subtle metabolic changes. While the Deauville scale provides a standardized framework, it may lack the sensitivity required to detect early or minimal changes in tumor metabolism. Clinically, these findings suggest that incorporating the ΔSUVmax method into routine interim PET/CT assessments could improve the accuracy of treatment response evaluations and long-term prognostication.^[21,22]^ This approach could facilitate more tailored treatment strategies, such as intensifying therapy for patients with suboptimal metabolic responses or de-escalating treatment for those with excellent responses, thus reducing toxicity and enhancing quality of life. Furthermore, the moderate agreement between the Deauville 5-point scale and the Lugano criteria (Kappa = 0.568) contrasts with the substantial agreement observed with the ΔSUVmax method (Kappa = 0.728), highlighting the need to integrate more quantitative methods into clinical practice.^[23,24]^ In our study, 2 nuclear medicine physicians independently assigned Deauville scores after a pre‐study calibration exercise, and discrepancies were resolved by a third senior reader. This uniform reading protocol and consensus adjudication helped minimize subjective variation. The formal Cohen kappa for interobserver agreement was 0.568 (*P* < .001), indicating substantial concordance. Although this level of agreement supports the reproducibility of Deauville scoring, residual variability can still arise from subtle differences in visual interpretation, particularly for borderline cases (e.g., uptake slightly above liver). In clinical practice, adherence to a standardized training protocol and periodic inter‐reader calibration are therefore recommended to preserve scoring consistency. Future multicenter studies should continue to monitor and report interobserver kappa values to ensure that Deauville remains a reliable tool across different institutions.

This study has several limitations. First, its retrospective, single‐center design may introduce selection bias and limit generalizability to other clinical settings with different protocols and patient populations. Second, although a pre‐study sample‐size calculation indicated that 60 patients provided adequate power for comparing Deauville and ΔSUVmax methods, the cohort remains relatively small; future studies should enroll larger, more diverse populations to confirm our findings. Third, the ΔSUVmax cutoff was derived from our own dataset via ROC curve analysis, but we were unable to perform external validation in an independent cohort; subsequent multicenter or prospective research should assess this threshold in separate populations to ensure its reliability. Fourth, our analysis was restricted to the Deauville 5-point scale and ΔSUVmax; head‐to‐head comparison with emerging imaging biomarkers (such as radiomic features, novel PET tracers, or metabolic tumor volume) was beyond this study’s scope. Future investigations should evaluate how ΔSUVmax performs relative to these quantitative markers and consider integration of molecular and genetic biomarkers to refine response assessment and prognostication in DLBCL.

## 5. Conclusions

The Deauville 5-point scale and ΔSUVmax method both demonstrated high value in evaluating the interim therapeutic response and predicting 12-month outcomes in DLBCL patients undergoing chemotherapy. Among these, the ΔSUVmax method showed higher accuracy. Integrating these methods into clinical practice can provide comprehensive insights into patient prognosis, aiding in the optimization of treatment strategies for better patient management.

## Acknowledgments

We appreciate the cooperation and informed consent provided by the patients for this study.

## Author contributions

**Conceptualization:** Jian-Ping Li, Pei Wang, Fu-Fu Liu, Kan Deng, Xin-Kai Wang.

**Data curation:** Jian-Ping Li, Pei Wang, Kan Deng, Xin-Kai Wang.

**Formal analysis:** Jian-Ping Li, Pei Wang, Fu-Fu Liu, Kan Deng, Xin-Kai Wang.

**Funding acquisition:** Xin-Kai Wang.

**Investigation:** Jian-Ping Li, Pei Wang, Fu-Fu Liu, Min Wu, Kan Deng.

**Methodology:** Jian-Ping Li, Pei Wang, Min Wu, Yong Wu.

**Resources:** Jian-Ping Li, Pei Wang, Fu-Fu Liu, Min Wu.

**Software:** Pei Wang, Min Wu.

**Writing – original draft:** Jian-Ping Li, Pei Wang.

**Writing – review & editing:** Yong Wu.
